# Denosumab in the Management of Glucocorticoid-Induced Osteoporosis: Long-Term Efficacy and Secondary Fracture Outcomes

**DOI:** 10.3390/jcm14051633

**Published:** 2025-02-27

**Authors:** Sian-Siang Liao, Ya-Lian Deng, Chiann-Yi Hsu, Hsu-Tung Lee, Chi-Ruei Li, Chi-Chan Yang

**Affiliations:** 1 Department of Neurosurgery, Neurological Institute, Taichung Veterans General Hospital, 1650 Taiwan Boulevard Sec. 4, Taichung 40705, Taiwan; sonic5407@gmail.com (S.-S.L.); leesd2001@hotmail.com (H.-T.L.); fantastic1694@gmail.com (C.-R.L.); 2 Department of Nursing, Taichung Veterans General Hospital, Taichung 40705, Taiwan; cfalsm@vghtc.gov.tw; 3 Department of Medical Research, Taichung Veterans General Hospital, Taichung 407405, Taiwan; chiann@vghtc.gov.tw; 4 Graduate Institute of Medical Sciences, National Defense Medical Center, Taipei 11490, Taiwan; 5 Department of Neurosurgery, Taichung Veterans General Hospital Puli Branch, Nantou 545402, Taiwan

**Keywords:** bone mineral density, denosumab, fracture, glucocorticoid, glucocorticoid-induced osteoporosis, osteoporosis

## Abstract

**Objectives**: Osteoporosis is a common complication in patients undergoing long-term corticosteroid therapy, particularly those with rheumatological and immunological conditions. Denosumab has shown potential in enhancing bone density and reducing fracture risk in such patients. This study evaluates the effectiveness of denosumab in osteoporosis management among corticosteroid-treated individuals. **Methods**: Between 2013 and 2022, 390 osteoporosis patients who received denosumab (60 mg subcutaneously every 6 months) for ≤18 months were enrolled. Patients were categorized based on corticosteroid use, and age-matching was applied to ensure comparability. Bone mineral density (BMD) and trabecular bone score (TBS) at the lumbar spine and femoral neck were assessed, and secondary fractures during the follow-up period were recorded. **Results**: Over the 18-month follow-up, both groups showed improvements in lumbar spine T-scores. The corticosteroid group increased from −2.1 ± 1.2 to −2.0 ± 1.3 (*p* < 0.001), while the non-corticosteroid group improved from −2.6 ± 1.2 to −2.4 ± 1.2 (*p* = 0.003). However, logistic regression analysis revealed that corticosteroid use remained a significant risk factor for secondary fractures (odds ratio: 1.69; 95% confidence interval: 1.11–2.56, *p* = 0.014), despite denosumab treatment. **Conclusions**: This retrospective study observed stabilization and a modest increase in BMD and TBS among corticosteroid users. Although differences in secondary fractures persisted between groups, denosumab shows potential for managing corticosteroid-induced osteoporosis. The study’s focus on Taiwanese patients limits its generalizability, and future research should include diverse populations to enhance applicability.

## 1. Introduction

Osteoporosis is a prevalent bone disorder that can result from primary or secondary causes. Primary osteoporosis, associated with aging and postmenopausal hormonal changes, leads to gradual bone loss over time [1]. In contrast, secondary osteoporosis arises due to underlying medical conditions or long-term medication use, with glucocorticoid-induced osteoporosis (GIOP) being the most common form [2]. GIOP is a significant concern for patients with rheumatological and immunological disorders who require prolonged glucocorticoid therapy. Chronic glucocorticoid use accelerates bone resorption and impairs bone formation, resulting in decreased bone mineral density and an increased risk of fractures. Vertebral fractures are the most characteristic feature of GIOP, but the risk of non-vertebral fractures, such as hip fractures, is also elevated [3]. Given its high prevalence and potential complications, the early recognition and management of GIOP are essential to prevent significant morbidity in affected patients.

The impact of glucocorticoid-induced osteoporosis has led to a substantial body of research focusing on pharmacological interventions for treating this condition. The primary treatment options include bisphosphonates, teriparatide, and denosumab. Bisphosphonates are widely recognized for their ability to inhibit bone resorption and are supported by numerous studies demonstrating their efficacy in reducing fracture risk in glucocorticoid-treated patients [4,5,6]. Teriparatide, a recombinant form of parathyroid hormone, has also been shown to increase bone mineral density and reduce fractures in these patients [7,8,9]. Denosumab, a human monoclonal antibody against the receptor activator of nuclear factor kappa-B ligand (RANKL), has emerged as a promising treatment for osteoporosis. It inhibits osteoclast formation, function, and survival, decreasing bone resorption and increasing bone density. While denosumab has shown substantial benefits in reducing fracture risk and increasing bone density in patients with postmenopausal osteoporosis [10,11], evidence supporting its efficacy in glucocorticoid-induced osteoporosis is less robust.

Despite denosumab’s promising results for the treatment of postmenopausal osteoporosis [12,13], there is a relative paucity of large-scale trials and strong evidence regarding its effectiveness, specifically in glucocorticoid-induced osteoporosis. Although limited, existing studies suggest that denosumab may benefit patients undergoing long-term glucocorticoid therapy by increasing bone density and potentially reducing the incidence of fractures [14]. However, more extensive and well-designed clinical trials are needed to establish denosumab’s long-term benefits and safety in this patient population.

Therefore, the present study aims to test the hypothesis that denosumab is effective in increasing bone mineral density and reducing fracture risk in osteoporosis patients also receiving long-term glucocorticoid. Specifically, this study seeks to answer the research question: “Does denosumab provide significant improvements in bone mineral density and fracture prevention compared to standard treatments in patients receiving long-term glucocorticoid therapy?” This investigation aims to fill the gap in current knowledge by providing more comprehensive data on the use of denosumab in osteoporosis patients also receiving long-term glucocorticoids, thereby aiding the development of more effective treatment protocols for this vulnerable patient group.

## 2. Methods

**Figure 1:**
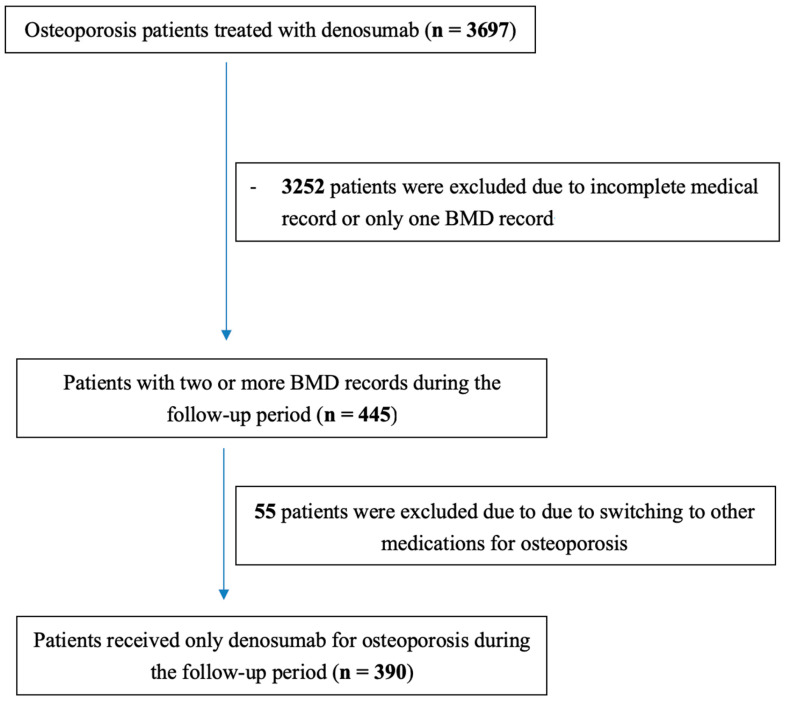
Flow diagram of inclusion and exclusion process.

To evaluate the effectiveness of denosumab in increasing bone mineral density and reducing fracture risk in patients with glucocorticoid-induced osteoporosis, we conducted a retrospective analysis in a single-institute study. Patient data were retrieved from hospital electronic registries for individuals who received denosumab between January 2013 and January 2022. The inclusion and exclusion criteria are illustrated in Figure 1. This study was approved on 21 January 2022, by the Taichung Veterans General Hospital Institutional Review Board (No. CF18067B-6) and was performed in accordance with the ethical standards as stated in the 1964 Declaration of Helsinki and its later amendments. Informed consent was waived because of the retrospective nature of the study and as the analysis used anonymous clinical data.

Patients were categorized into two groups: those receiving glucocorticoid treatment and those not receiving glucocorticoids. To assess potential factors influencing bone mineral density (BMD), demographic data (age, sex) and laboratory parameters (albumin, creatinine, serum calcium, phosphate, and intact parathyroid hormone) were analyzed. Additionally, cumulative glucocorticoid dosage was documented.

The BMD of the lumbar spine (L1–L4) and bilateral femoral neck were measured using dual-energy X-ray absorptiometry (DXA) (Lunar iDXA; GE HealthCare, Madison, WI, USA) at baseline and at 12 months. Lumbar spine BMD was specifically assessed at L1–L4 levels following standard DXA scanning protocols. The trabecular bone score (TBS) was evaluated using TBS iNsight^®^ software (v1.9, Medimaps, Merignac, France), which is integrated with the DXA system. TBS values were derived from DXA scans and calculated as the mean TBS of L1–L4 vertebrae.

At baseline, before the first administration of denosumab, BMD and TBS were measured and later compared with follow-up BMD data at 12 months. The primary outcomes assessed were BMD and TBS at the lumbar spine and femoral neck. Additionally, secondary fracture events occurring within 18 months after the first denosumab administration were recorded.

### Statistical Analysis

Categorical variables were analyzed using the chi-square test, while continuous variables were assessed using the Mann–Whitney U test. Participants were classified into two groups based on glucocorticoid treatment. The with-glucocorticoid group comprised patients who had received glucocorticoid therapy for at least 270 days before their first dose of denosumab, with a daily dosage exceeding 5 mg. Additionally, glucocorticoid use had to continue for at least 270 days following the administration of the first denosumab dose. All other participants were categorized into the without-glucocorticoid group.

Initially, the two groups showed discrepancies in age and number of participants. The with-glucocorticoid group comprised 122 individuals, whereas the without-glucocorticoid group included 268 individuals. The demographic characteristics of the cohort are presented in Table 1. To create comparable groups differing only in glucocorticoid exposure, we performed 1:1 matching based on age, with a matching tolerance of ±0.4 years. Each control was used only once (i.e., no replacement was allowed within the case–control groups). Additionally, we accounted for potential confounders by ensuring that baseline characteristics, such as lumbar BMD, TBS, and relevant laboratory values (e.g., serum creatinine), were balanced between the groups.

Primary outcomes, including follow-up BMD and TBS, measured in the lumbar spine and femoral neck, were analyzed in these two groups. The secondary outcomes of subsequent fractures were analyzed using conditional logistic regression to identify possible causative factors. All continuous variables are presented as the mean ± standard deviation. Statistical significance was set at *p* = 0.05.

## 3. Results

Between 2013 and 2022, a total of 390 patients with osteoporosis who received denosumab treatment for up to 18 months were enrolled in this study. Denosumab (60 mg) was administered subcutaneously every six months. The demographic characteristics of the patients are presented in Table 1. Significant differences were observed between the “without-glucocorticoid” and “with-glucocorticoid” groups. Patients in the “with-glucocorticoid” group were younger, with a mean age of 67.2 ± 10.6 years, compared to 70.5 ± 10.3 years in the “without-glucocorticoid” group (*p* = 0.006). Furthermore, elevated serum creatinine levels were noted (1.5 ± 2.3 mg/dL vs. 1.2 ± 1.7 mg/dL, *p* = 0.003). Interestingly, baseline spinal bone mineral density (BMD) and T-score were significantly better in the “with-glucocorticoid” group, with a spinal BMD of 0.88 ± 0.15 compared to 0.85 ± 0.15 in the “without-glucocorticoid” group (*p* = 0.009), and a spinal T-score of −2.02 ± 1.17 versus −2.36 ± 1.25, respectively (*p* = 0.003).

Considering the age discrepancy between the two groups as a potential confounder, we performed 1:1 matching based on age to create comparable groups differing only in glucocorticoid exposure, with a matching tolerance of ±0.4 years. Paired *t*-tests confirmed that there were no significant differences in age between the matched groups (*p* = 0.997) (Table 2). Despite matching, significant differences in baseline spinal BMD, and T-score persisted.

After an 18-month follow-up period, the “with-glucocorticoid” group exhibited significantly higher spinal BMD compared to the “without-glucocorticoid” group (0.91 ± 0.16 vs. 0.86 ± 0.14, *p* = 0.048). However, BMD at other skeletal sites tended to be lower in the “with-glucocorticoid” group than in the “without-glucocorticoid” group (Table 3). Both groups demonstrated improvements in lumbar spine BMD and T-scores over time (Table 4). Specifically, in the glucocorticoid group, spinal BMD increased from 0.87 ± 0.14 to 0.90 ± 0.15, while the T-score improved from −2.1 ± 1.2 to −2.0 ± 1.3. Similarly, in the non-glucocorticoid group, spinal BMD increased from 0.82 ± 0.14 to 0.86 ± 0.14, with a corresponding improvement in the T-score from −2.6 ± 1.2 to −2.4 ± 1.2.

**Figure 2:**
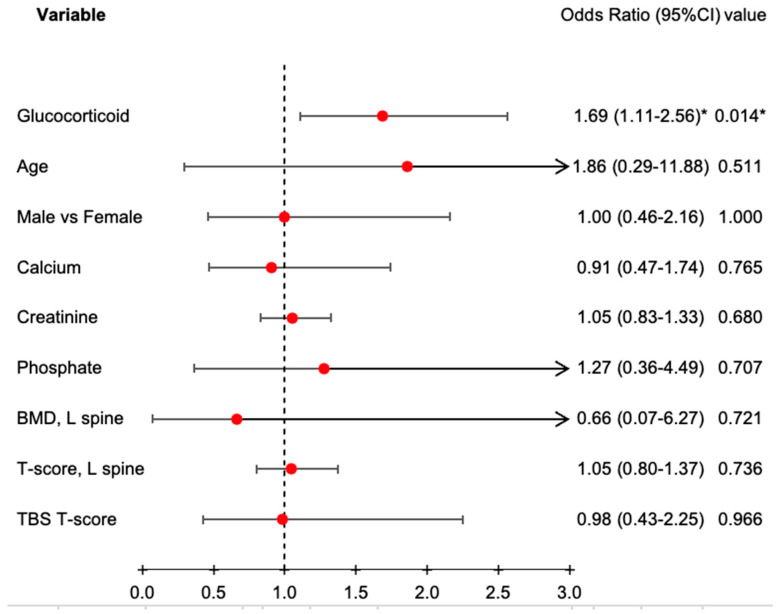
Conditional logistic regression for secondary fracture analysis. Logistic regression analysis for secondary fractures revealed that only glucocorticoid administration remained a significant influencing factor (odds ratio: 1.69; 95% confidence interval: 1.11–2.56); *p* = 0.014; * *p* < 0.05.

Regarding secondary fractures, the “with-glucocorticoid” group had a significantly higher re-fracture rate compared to the “without-glucocorticoid” group (55.1% vs. 32.7%, *p* < 0.001) (Table 5). However, when analyzing fracture sites, no significant differences were observed between the two groups. Additionally, we found significant differences in glucocorticoid use (40.0% vs. 62.8%), age (66.7 ± 8.1 years vs. 71.2 ± 7.8 years), and baseline serum albumin levels (4.1 ± 0.4 mg/dL vs. 3.9 ± 0.5 mg/dL) between the “non-fracture” and “fracture” groups (Table 6). However, logistic regression analysis for secondary fractures revealed that glucocorticoid administration remained a significant influencing factor (odds ratio: 1.69; 95% confidence interval: 1.11—2.56, *p* = 0.014) despite the administration of denosumab (Figure 2).

## 4. Discussion

This 18-month retrospective observational study provides additional evidence of the efficacy of denosumab in glucocorticoid-treated individuals at a high risk of fracture. The beneficial effect of denosumab on the BMD of the spine and hip and the T-score of the spine was observed in the primary outcome analysis for both the glucocorticoid and non-glucocorticoid groups. Furthermore, after denosumab administration, the follow-up T-score and TBS data in the glucocorticoid group were not inferior to those in the non-glucocorticoid group. However, when discussing secondary fractures, glucocorticoids remained an influencing factor despite both groups showing significant improvements in BMD and T-scores with denosumab treatment.

Compared with postmenopausal osteoporosis, evidence supporting the efficacy of denosumab in the treatment of glucocorticoid-induced osteoporosis is considerably less extensive. Since its introduction and FDA approval in 2010, denosumab has been well documented as a feasible treatment for postmenopausal women with osteoporosis who are at a high risk of fracture [15,16,17,18,19,20]. By blocking the RANKL pathway, denosumab effectively inhibits osteoclast activity. However, in the context of glucocorticoid-induced osteoporosis, which involves both the inhibition of osteoblast function and the direct stimulation of osteoclasts by glucocorticoids [21,22,23,24,25,26,27], further evidence is required to substantiate the benefits of denosumab.

Established guidelines recommend bisphosphonates as the first-line treatment for the prevention and management of GIOP in most patients [28,29]. However, denosumab serves as an alternative treatment option for individuals who cannot tolerate bisphosphonates or in cases where bisphosphonates prove ineffective. A randomized, open-label study by Mok et al. [30], conducted on patients receiving long-term prednisolone therapy (≥2.5 mg/day for ≥1 year), demonstrated that spinal bone mineral density (BMD) at 12 months was significantly higher in the denosumab group compared to the bisphosphonate group. In such clinical scenarios, bone turnover markers, including β-isomerized C-terminal telopeptides (β-CTx), and procollagen type 1 amino-terminal propeptide (P1NP), serve as crucial indicators of bone homeostasis [31,32]. Notably, these markers were more strongly suppressed by denosumab compared to bisphosphonates. Similar findings were reported by Iseri et al. in 2018 [33], who found that denosumab led to a greater increase in spinal BMD compared to alendronate at the 12-month follow-up. A recent multicenter randomized, double-blind trial also showed that denosumab was more effective than risedronate in patients who started or continued glucocorticoid therapy [34]. Furthermore, recent meta-analyses provide positive evidence comparing the treatment effects of denosumab and bisphosphonates [11,35,36].

In our present study, although we observed stabilization and a slight improvement in BMD and TBS among glucocorticoid-treated participants, suggesting denosumab’s potential against glucocorticoid-induced osteoporosis, secondary fracture analysis revealed that glucocorticoid administration remained a significant influencing factor (odds ratio: 1.69; 95% confidence interval: 1.11–2.56, *p* = 0.014), despite the administration of denosumab. This finding may be explained by the fact that glucocorticoids negatively affect bone by decreasing bone formation through reduced osteoblast production and activity [37]. Chiodini et al. raised concerns regarding the long-term efficacy of denosumab in glucocorticoid-induced osteoporosis, suggesting that it might be less effective over extended periods [38]. Furthermore, short-term or intermittent use of denosumab likely provides no net benefit, as discontinuation is associated with rapid bone loss and an increased risk of multiple vertebral fractures [39,40]. It could also be implied that denosumab provided an additional approximate gain of 1% in BMD over 12 to 24 months; however, the increase in BMD over a short period was not large enough to reduce fracture incidence [41]. Therefore, it remains unclear whether denosumab offers sufficient benefits in patients with low bone turnover due to the long-term use of glucocorticoids. While future prospective studies in postmenopausal women are required to confirm these findings, our results offer a cautionary note for postmenopausal women undergoing osteoporosis treatment with Prolia and who are also on long-term steroid therapy. Although denosumab can significantly improve BMD, this improvement does not necessarily translate into a reduced risk of secondary fractures in this population.

There are several limitations to our study. First, this was a retrospective and observational study. Second, the follow-up period of 18 months may have been insufficient to fully assess the drug’s efficacy in reducing fragility fractures. Third, recent clinical evidence indicates that denosumab may be positively associated with glycemic parameters [42], which could also be influenced by concurrent glucocorticoid use. The absence of detailed glycemic data from our cohort may obscure its potential benefits in preventing glucocorticoid-induced hyperglycemia or diabetes. Fourth, our study cohort consisted solely of Taiwanese individuals. To gain a more comprehensive understanding of denosumab use, it is essential to consider a broader perspective that includes epidemiological, genetic, and epigenetic factors [43]. Consequently, the generalizability of our findings to other racial and ethnic populations, as well as to different geographical regions, remains uncertain and requires further investigation.

## 5. Conclusions

In our 18-month retrospective study, we noted a stabilization and slight improvement in BMD and TBS in glucocorticoid-treated patients, suggesting the potential of denosumab against glucocorticoid-induced osteoporosis. However, the data did not provide strong evidence to support the effectiveness of denosumab in preventing secondary compression fractures in these patients. Therefore, clinicians and patients should be aware that the benefits of BMD enhancement with denosumab may not extend to the effective prevention of secondary fractures in postmenopausal women receiving long-term steroid treatment.

## Figures and Tables

**Table 1:** Demographic data of cohort.

	Without Glucocorticoid (n = 268)		With Glucocorticoid (n = 122)		*p* Value
	Mean	±SD	Mean	±SD	
Age	70.5	±10.3	67.2	±10.6	0.006 **
Sex, n (%)					0.329
Female	226	(84.3%)	98	(80.3%)	
Male	42	(15.7%)	24	(19.7%)	
Albumin	4.1	±0.5	4.0	±0.5	0.044
ALP	110.3	±72.0	124.6	±140.7	0.731
Serum Calcium	9.0	±0.6	9.0	±0.6	0.920
Serum Creatinine	1.2	±1.7	1.5	±2.3	0.003 **
Serum iPTH	201.1	±578.0	340.0	±735.9	0.131
Serum Phosphate	3.8	±0.8	4.0	±1.2	0.580
Baseline BMD and TBS					
BMD, L spine	0.85	±0.15	0.88	±0.15	0.009 **
BMD, left femoral neck	0.67	±0.11	0.68	±0.10	0.229
BMD, right femoral neck	0.67	±0.09	0.66	±0.10	0.831
T-score, L spine	−2.36	±1.25	−2.02	±1.17	0.003 **
T-score, left femoral neck	−2.38	±0.80	−2.30	±0.81	0.254
T-score, right femoral neck	−2.37	±0.84	−2.43	±0.81	0.650
TBS BMD	1.24	±0.10	1.26	±0.10	0.489
TBS T-score	−2.34	±1.17	−2.23	±1.10	0.832

ALP, alkaline phosphatase; BMD, bone mineral density; iPTH, intact parathyroid hormone; L spine: lumbar spine; TBS, trabecular bone score; SD, standard deviation. ** *p* < 0.01.

**Table 2:** Demographic data after matching.

	Without Glucocorticoid (n = 107)		With Glucocorticoid (n = 107)		*p* Value
	Mean	±SD	Mean	±SD	
Age	68.6	±8.3	68.6	±8.3	0.997
Sex, n (%)					0.579
Female	91	(85.0%)	88	(82.2%)	
Male	16	(15.0%)	19	(17.8%)	
Albumin	4.1	±0.4	4.0	±0.5	0.207
ALP	112.0	±71.1	109.9	±87.9	0.559
Serum Calcium	9.1	±0.6	9.0	±0.6	0.449
Serum Creatinine	1.3	±2.1	1.4	±2.2	0.039 *
Serum iPTH	181.6	±361.6	296.3	±694.9	0.314
Serum Phosphate	3.9	±0.6	4.0	±1.2	0.429
Baseline BMD and TBS					
BMD, L spine	0.83	±0.15	0.87	±0.15	0.015 *
BMD, left femoral neck	0.66	±0.11	0.67	±0.10	0.610
BMD, right femoral neck	0.67	±0.09	0.65	±0.10	0.093
T-score, L spine	−2.52	±1.25	−2.05	±1.20	0.005 *
T-score, left femoral neck	−2.34	±0.74	−2.34	±0.79	0.662
T-score, right femoral neck	−2.33	±0.76	−2.48	±0.80	0.117
TBS BMD	1.22	±0.10	1.25	±0.10	0.366
TBS T-score	−2.47	±1.09	−2.34	±1.05	0.609

ALP, alkaline phosphatase; BMD, bone mineral density; iPTH, intact parathyroid hormone; L spine: lumbar spine; TBS, trabecular bone score; SD, standard deviation. * *p* < 0.05.

**Table 3:** Follow-up data of BMD and TBS.

	Without Glucocorticoid (n = 107)		With Glucocorticoid (n = 107)		*p* Value
	Mean	±SD	Mean	±SD	
Follow-up BMD and TBS					
BMD, L spine	0.86	±0.14	0.91	±0.16	0.048 *
BMD, left femoral neck	0.69	±0.09	0.68	±0.10	0.903
BMD, right femoral neck	0.68	±0.09	0.66	±0.09	0.378
T-score, L spine	−2.35	±1.18	−1.94	±1.33	0.055
T-score, left femoral neck	−2.31	±0.70	−2.34	±0.81	0.878
T-score, right femoral neck	−2.38	±0.73	−2.47	±0.74	0.403
TBS BMD	1.24	±0.09	1.25	±0.10	0.601
TBS T-score	−2.44	±1.05	−2.19	±1.46	0.584

BMD, bone mineral density; L spine: lumbar spine; TBS, trabecular bone score; SD, standard deviation. * *p* < 0.05.

**Table 4:** BMD, T-score, and TBS analysis after denosumab treatment.

	Without Glucocorticoid					With Glucocorticoid				
	Before		After		*p* Value	Before		After		*p* Value
	Mean	±SD	Mean	±SD		Mean	±SD	Mean	±SD	
BMD										
L spine	0.82	±0.14	0.86	±0.14	<0.001 **	0.87	±0.14	0.90	±0.15	<0.001 **
Left femoral neck	0.67	±0.09	0.69	±0.09	<0.001 **	0.68	±0.09	0.68	±0.10	0.972
Right femoral neck	0.67	±0.09	0.68	±0.09	0.161	0.66	±0.10	0.67	±0.09	0.010 *
T-score										
L spine	−2.6	±1.2	−2.4	±1.2	0.003 **	−2.1	±1.2	−2.0	±1.3	0.002 **
Left femoral neck	−2.3	±0.7	−2.3	±0.7	0.118	−2.3	±0.7	−2.3	±0.8	0.039 *
Right femoral neck	−2.3	±0.8	−2.4	±0.7	0.540	−2.4	±0.8	−2.5	±0.7	0.804
TBS										
BMD	1.22	±0.11	1.22	±0.09	0.991	1.25	±0.10	1.27	±0.11	0.086
T-score	−2.5	±1.1	−2.5	±0.9	0.809	−2.3	±1.1	−1.9	±1.6	0.085

BMD, bone mineral density; L spine: lumbar spine; TBS, trabecular bone score; SD, standard deviation. * *p* < 0.05. ** *p* < 0.01.

**Table 5:** Re-fracture rate comparison between groups.

	Without Glucocorticoid (n = 107)		With Glucocorticoid (n = 107)		*p* Value
	Mean	±SD	Mean	±SD	
Re-fracture, n (%)					
Total	35	(32.7%)	59	(55.1%)	<0.001 **
Femur	16	(45.7%)	30	(50.8%)	0.630
Radius	3	(8.6%)	1	(1.7%)	0.144
Spine	17	(48.6%)	29	(49.2%)	0.957

SD, standard deviation. ** *p* < 0.01.

**Table 6:** Analysis of secondary fractures.

	Non-Fracture (n = 120)		Fracture (n = 94)		*p* Value
	Mean	±SD	Mean	±SD	
Patients receive glucocorticoid, n (%)	48	(40.0%)	59	(62.8%)	0.001 **
Age	66.7	±8.1	71.2	±7.8	<0.001 **
Sex, n (%)					0.816
Female	101	(84.2%)	78	(83.0%)	
Male	19	(15.8%)	16	(17.0%)	
Albumin	4.1	±0.4	3.9	±0.5	0.002 **
ALP	112.8	±77.9	108.1	±85.2	0.357
CALCIUM	9.1	±0.6	8.9	±0.6	0.236
Creatinine	1.5	±2.3	1.2	±1.8	0.808
iPTH	228.2	±391.7	272.1	±809.5	0.644
Phosphate	3.9	±0.8	4.1	±1.3	0.534
Baseline					
BMD, L spine	0.8	±0.2	0.9	±0.1	0.751
BMD, left femoral neck	0.7	±0.1	0.7	±0.1	0.229
BMD, right femoral neck	0.7	±0.1	0.6	±0.1	0.037 *
T-score, L spine	−2.3	±1.3	−2.2	±1.2	0.203
T-score, left femoral neck	−2.3	±0.7	−2.4	±0.8	0.847
T-score, right femoral neck	−2.4	±0.7	−2.5	±0.9	0.476
TBS BMD	1.2	±0.1	1.3	±0.1	0.310
TBS T-score	−2.5	±1.0	−2.1	±1.3	0.362
After					
BMD, L spine	0.9	±0.1	0.9	±0.2	0.872
BMD, left femoral neck	0.7	±0.1	0.7	±0.1	0.605
BMD, right femoral neck	0.7	±0.1	0.7	±0.1	0.028 *
T-score, L spine	−2.2	±1.2	−2.1	±1.3	0.329
T-score, left femoral neck	−2.3	±0.7	−2.3	±0.8	0.374
T-score, right femoral neck	−2.4	±0.7	−2.5	±0.8	0.344
TBS BMD	1.2	±0.1	1.3	±0.1	0.626
TBS T-score	−2.4	±1.1	−2.0	±1.7	0.399

ALP, alkaline phosphatase; BMD, bone mineral density; iPTH, intact parathyroid hormone; L spine: lumbar spine; TBS, trabecular bone score; SD, standard deviation. * *p* < 0.05. ** *p* < 0.01.

## Data Availability

Data are available upon reasonable request. The datasets used during the current study are available from the Taichung Veterans General Hospital; however, restrictions apply regarding the availability of these data, as they are not publicly available. However, the data are available from the corresponding author upon reasonable request and with permission from the Taichung Veterans General Hospital.
